# Extraversion and neuroticism relate to topological properties of resting-state brain networks

**DOI:** 10.3389/fnhum.2013.00257

**Published:** 2013-06-11

**Authors:** Qing Gao, Qiang Xu, Xujun Duan, Wei Liao, Jurong Ding, Zhiqiang Zhang, Yuan Li, Guangming Lu, Huafu Chen

**Affiliations:** ^1^School of Mathematical Sciences, University of Electronic Science and Technology of ChinaChengdu, China; ^2^Key Laboratory for Neuroinformation of Ministry of Education, School of Life Science and Technology, University of Electronic Science and Technology of ChinaChengdu, China; ^3^Department of Medical Imaging, Jinling Hospital, Clinical School, Medical College, Nanjing UniversityNanjing, Jiangsu, China; ^4^School of Politics and Public Managements, University of Electronic Science and Technology of ChinaChengdu, China

**Keywords:** resting-state, functional magnetic resonance imaging, graph topological properties, extraversion, neuroticism

## Abstract

With the advent and development of modern neuroimaging techniques, there is an increasing interest in linking extraversion and neuroticism to anatomical and functional brain markers. Here, we aimed to test the theoretically derived biological personality model as proposed by Eysenck using graph theoretical analyses. Specifically, the association between the topological organization of whole-brain functional networks and extraversion/neuroticism was explored. To construct functional brain networks, functional connectivity among 90 brain regions was measured by temporal correlation using resting-state functional magnetic resonance imaging (fMRI) data of 71 healthy subjects. Graph theoretical analysis revealed a positive association of extraversion scores and normalized clustering coefficient values. These results suggested a more clustered configuration in brain networks of individuals high in extraversion, which could imply a higher arousal threshold and higher levels of arousal tolerance in the cortex of extraverts. On a local network level, we observed that a specific nodal measure, i.e., betweenness centrality (BC), was positively associated with neuroticism scores in the right precentral gyrus (PreCG), right caudate nucleus, right olfactory cortex, and bilateral amygdala. For individuals high in neuroticism, these results suggested a more frequent participation of these specific regions in information transition within the brain network and, in turn, may partly explain greater regional activation levels and lower arousal thresholds in these regions. In contrast, extraversion scores were positively correlated with BC in the right insula, while negatively correlated with BC in the bilateral middle temporal gyrus (MTG), indicating that the relationship between extraversion and regional arousal is not as simple as proposed by Eysenck.

## Introduction

In Eysenck's personality theory, he proposed three fundamental dimensions of personality: extraversion, neuroticism, and psychoticism (Eysenck, 1967; Eysenck and Eysenck, 1985). It is now acknowledged that extraversion and neuroticism have their biological bases (Matthews and Gilliland, 1999), while the neuropsychology of the third dimension, psychoticism, has not been worked out in detail. The arousal theory of Eysenck (1967) related extraversion to arousability of the reticulo-cortical circuit and proposed a higher arousal threshold in cortex and higher levels of arousal tolerance in extraverts (Eysenck, 1967; Eysenck and Eysenck, 1985; Fischer et al., 1998). Neuroticism, on the other hand, is associated with arousability of the limbic circuit, such that individuals with higher neuroticism scores have greater activation levels and lower thresholds within subcortical structures (Eysenck, 1990; Wei et al., 2012).

With the advent and development of modern neuroimaging techniques, there is increasing interest in exploring neuroanatomical or neurofunctional correlates of extraversion and neuroticism, to test the theoretically proposed biological explanation of the two fundamental dimensions. Neuroanatomical studies have found extraversion was associated with structural/anatomic variations in the middle and inferior frontal regions, fusiform gyrus, and insula (INS), whereas neuroticism was associated with variations in the orbitofrontal cortex, precentral gyrus (PreCG), and amygdala (AMYG) (Rauch et al., 2005; Omura et al., 2005b; Wright et al., 2006, 2007; Sollberger et al., 2009; DeYoung, 2010). In neurofunctional studies, functional magnetic resonance imaging (fMRI) experiments have also demonstrated that specific brain regions that are engaged during cognitive-affective tasks were associated with specific personality dimensions. For example, activations in the prefrontal cortex, parietal cortex, anterior cingulated cortex (ACC), and middle temporal gyrus (MTG) were correlated with extraversion (Canli et al., 2001; Eisenberger et al., 2005; Hutcherson et al., 2008; Tamura et al., 2012), while activations in the frontal cortex, dorsomedial prefrontal cortex, and AMYG were related to neuroticism (Canli et al., 2001; Haas et al., 2007; Hooker et al., 2008; Harenski et al., 2009). In addition, a positron emission tomography (PET) study assessing resting regional cerebral blood flow (rCBF) found that regions in ACC and temporal lobes were correlated with extraversion (Johnson et al., 1999). Extraversion was associated with regional cerebral glucose metabolism (rCMRglu) assessed by PET in right putamen, while neuroticism was associated with rCMRglu in the medial prefrontal cortex (MPFC) (Kim et al., 2008). These studies indicated specialized, spatially distributed regions were associated with personality dimensions of extraversion and neuroticism, respectively, and provided neurobiological evidence for the hypothesized biological model of Eysenck's personality.

Instead of detection activation paradigms by task-based fMRI, resting-state fMRI studies observe intrinsic spontaneous fluctuations in the blood oxygen level-dependent (BOLD) fMRI signal while avoiding the constraints of task-based approaches (Raichle et al., 2001; Fox and Raichle, 2007; Raichle and Snyder, 2007; Adelstein et al., 2011). There is accumulating evidence for local characteristics of resting brain functions associated with personality dimensions using resting-state fMRI (Kunisato et al., 2011; Wei et al., 2011, 2012; Hahn et al., 2012). Using regional homogeneity (ReHo) approach, our prior study found ReHo was correlated negatively with extraversion in the MPFC, and correlated positively in INS, cerebellum, and cingulate gyrus; whereas neuroticism had negative correlation with ReHo in left middle frontal gyrus (Wei et al., 2011). In addition, by using other local characteristics, i.e., the fractional amplitude of low-frequency fluctuations (fALFF), our previous study found positive correlations between LFF amplitude at Slow-5 and extraversion in MPFC and PCU, and between LFF amplitude at Slow-5 and neuroticism in right PreCG; LFF amplitude at Slow-4 was negatively associated with extraversion and neuroticism in left hippocampus (HIP) and bilateral superior temporal cortex (STC), respectively (Wei et al., 2012). Table 1 summarizes the main results on the characteristics of resting brain functions associated with extraversion and neuroticism in recent resting-state fMRI studies.

**Table 1:** **The main results on the characteristics of resting brain functions associated with extraversion and neuroticism in recent resting-state fMRI studies**.

**Method**	**fALFF (Kunisato et al., 2011)**	**Seed-based (ACC and PCU) FC (Adelstein et al., 2011)**	**ReHo (Wei et al., 2011)**	**fALFF (Slow-5 and Slow-4 bands) (Wei et al., 2012)**
Extraversion	Striatum PCU	FC between seed regions and lateral paralimbic regions	INS	MPFC and PCU at Slow-5
	SFG		MCG	
			MPFC	HIP at Slow-4
			MTG	
			Cerebellum	
Neuroticism	MFG	FC between seed regions and the dorsomedial prefrontal cortex	MFG	PreCG at Slow-5
	PCU			STC at Slow-4

ACC, anterior cingulate cortex; fALFF, fractional amplitude of low-frequency fluctuations; FC, functional connectivity; HIP, hippocampus; INS, insula; MCG, middle cingulate gyrus; MFG, middle frontal gyrus; MPFC, medial prefrontal cortex; MTG, middle temporal gyrus; PCU, precuneus; PreCG, precentral gyrus; ReHo, regional homogeneity; SFG, superior frontal gyrus; STC, superior temporal cortex.

From a functional integration perspective in the human brain, the multiple spatially distinct brain regions are functionally connected with coherent temporal dynamics (Friston et al., 1997; Sporns et al., 2000; Van Den Heuvel et al., 2009), making up complex and reciprocal brain networks even when we are at rest (Greicius et al., 2003; Damoiseaux et al., 2006; Van Den Heuvel et al., 2009). Such networks are thought to provide the physiological basis for information processing and mental representation (Canli, 2004; Bullmore and Sporns, 2009). Furthermore, evidence for small-world attributes of brain networks has been reported in the relative studies (Sporns et al., 2004; Stam, 2004; Eguiluz et al., 2005; Achard et al., 2006; Van Den Heuvel et al., 2009), indicating that small-world architectures in brain networks deviating from randomness reflect their specific functionality (Watts and Strogatz, 1998; Latora and Marchiori, 2001; Stam and Reijneveld, 2007; Bullmore and Sporns, 2009; Van Den Heuvel et al., 2009; He and Evans, 2010). Since personality factors may well be related to the networks in the brain (Canli, 2004; Wilt and Revelle, 2009), the analysis of task-independent, resting-state functional connectivity may reveal the intrinsically organized functional brain networks (Biswal et al., 1995), and allow for a better understanding of the neurobiological bases of extraversion and neuroticism. The recent study by Adelstein et al. (2011) found that extraversion and neuroticism were encoded within resting-state functional connectivity between seed regions and the lateral paralimbic regions and dorsomedial prefrontal cortex, respectively (Adelstein et al., 2011). However, the study was seed-based and lacked a network perspective on brain dynamics. In the present study, we hypothesized that the topological organization of the whole-brain functional networks would be associated with inter-individual variations in extraversion and neuroticism, and would link to Eysenck's cortical arousal theory of the two dimensions. To test our hypothesis, an exploratory analysis based on graph theory was thereby performed on the resting-state fMRI data of 71 healthy subjects, to detect the intrinsic resting-state functional connectivity mechanisms underlying the two personality dimensions.

## Materials and methods

### Participants

We conducted the analysis with the same dataset in our previous study (Wei et al., 2011). Eighty-seven healthy right-handed subjects (48 males; age range: 17–36 yrs, mean age: 23.5 yrs) with no history of neurological or psychiatric disorders participated in the study. The present study was approved by the local Medical Ethics Committee at Jinling Hospital, Nanjing University School of Medicine, and the informed written consents were obtained from all participants.

### Personality questionnaires

The revised Eysenck personality questionnaire short scale for Chinese (EPQ-RSC) (Eysenck, 1991; Qian et al., 2000) was used to assess personality dimensions of extraversion, neuroticism, and psychoticism of each subject before MRI scanning. Raw scores of the three dimensions were then converted into *T*-scores using the formula (Qian et al., 2000), respectively:

$$T=50+10\times \frac{\text{raw score}-\text{mean}}{\text{SD}},$$

where *mean* represents the mean value of the personality scores over all the subjects; *SD* is the standard deviation of the personality scores. We focused our analyses on extraversion and neuroticism whose resultant *T*-scores were used for calculating correlations with the brain network metrics.

### Image acquisition

Resting-state fMRI images were acquired using a single-shot, gradient-recalled echo planar imaging (EPI) sequence on a 3.0-T Siemens Trio scanner (Jinling Hospital, Nanjing, China). The acquisition parameters were: *TR* = 2000 ms, *TE* = 30 ms, field of view (FOV) = 240 mm, image matrix size = 64 × 64, voxel size = 3.75 × 3.75 × 4 mm^3^, 30 transverse slices without slice gap, flip angle = 90°, and a total of 255 volumes for each subject.

### Data preprocessing

Data preprocessing was performed using the Statistical Parametric Mapping software (SPM8, http://www.fil.ion.ucl.ac.uk/spm). The first five volumes were discarded to ensure steady-state longitudinal magnetization. The remaining resting-state fMRI images were first corrected by the acquisition time delay among different slices, and then realigned to the first volume for head-motion correction. The dataset with translational or rotational parameters exceeding ±1 mm or ±1° would be excluded, according to our previous study on functional connectivity network (Liao et al., 2010). The images of remaining 71 participants were further spatially normalized into a standard stereotaxic space at 3 × 3 × 3 mm^3^, using the Montreal Neurological Institute (MNI) template in SPM8. In order to avoid artificially introducing local spatial correlation, no spatial smoothing was applied, as previous studies suggested (Salvador et al., 2005; Achard et al., 2006; Achard and Bullmore, 2007; Liao et al., 2010). Since recent studies have showed that functional connectivity analysis is sensitive to gross head motion effects (Power et al., 2012; Van Dijk et al., 2012), we further evaluated the framewise displacement (FD) (Power et al., 2012) to express instantaneous head motion, and the threshold of 0.5 was suggested. The *mean* ± *SD* of FD over subjects was: 0.1080 ± 0.0159. Six subjects' FD values were beyond 0.5, but only in one frame for each subject. Scrubbing process was performed using toolbox “ArtRepair” in SPM8.

The mean time series of each ROI was corrected by a linear regression to remove the possible spurious variances including six head motion parameters acquired from the SPM8 preprocessing, the white matter (WM) and the ventricular brain signals averaged from a WM mask and a ventricular mask respectively (Fox et al., 2005; Salvador et al., 2005; Tian et al., 2006; Liao et al., 2010). The residuals of these regressions were temporally band-pass filtered (0.01 < *f* < 0.08 Hz) to reduce low-frequency drifts and physiological high-frequency respiratory and cardiac noise (Biswal et al., 1995), and linearly detrended for further functional connectivity and graph-theory analysis (Tian et al., 2006; Liao et al., 2010). The following approaches based on graph theory were performed by an in-house program coded in MATLAB (The Mathworks, Natick, MA).

### Computation of functional connectivity network

#### Node definition

To define the brain nodes, the anatomical parcellation was performed using the automated anatomical labeling (AAL) template, segmenting the images into 90 anatomical regions of interests (ROIs) (45 ROIs for each hemisphere). The representative time series in each ROI was obtained by averaging the fMRI time series across all voxels in the ROI.

#### Edge definition

To define the network edges, the residuals of the regression analysis were used to compute the Pearson's correlation, resulting in a 90 × 90 correlation matrix for each subject. A Fisher's *r*-to-*z* transformation was applied to the correlation matrices of all the subjects to improve the normality of the correlation coefficients (*r*) (Liu et al., 2008). The undirected edge *e*_*ij*_ between node *i* and node *j* is defined as:

$$e_{ij}=\{\begin{array}{ll} 1 & \text{when}|r_{ij}|>T\\ 0 & \text{otherwise} \end{array}$$

In general, if the absolute value of *r*_*ij*_ of a pair of brain regions, *i* and *j*, exceeds a predefined threshold *T*, an edge is assumed to exist; otherwise, no existence would be assumed (Liao et al., 2010).

### Graph theoretical analysis

#### Network metrics

The topological properties of the brain functional networks can be measured by both nodal and global network measures. In this study, we calculated the nodal measures including the degree *K*_*i*_, the clustering coefficient *C*_*i*_, the minimum path length *L*_*i*_, the efficiency *E*_*i*_, and the betweenness centrality *BC*_*i*_ of a node *i*; the global measures including the average degree *K*, the network efficiency involving the local efficiency *E*_local_ and the global efficiency *E*_global_, the characteristic path length *L*, the clustering coefficient of a network *C*, the normalized clustering coefficient γ, the normalized characteristic path length λ, and the small-worldness σ.

***Degree.*** The degree at each node, *K*_*i*_, *i* = 1, 2,…, 90, is defined as the number of nodes in a subgraph *G*_*i*_, which is the graph including the nodes that are direct neighbors of node *i*. Briefly, *K*_*i*_ denotes to which extent the node is connected to the rest of the network (Bullmore and Sporns, 2009; Wang et al., 2010). A node with a higher degree has more connections (where each connection is counted once). The average degree *K* is the mean of *K*_*i*_ of all the nodes in the network.

***Clustering coefficient.*** The absolute clustering coefficient *C*_*i*_ of a node is the ratio between the number of existing connections and the number of all possible connections in the subgraph *G*_*i*_. *C*_*i*_ quantifies the level of local connectedness within a network (Bullmore and Sporns, 2009; Van Den Heuvel et al., 2009; He and Evans, 2010)

$$C_{i}=\frac{e_{i}}{K_{i}(K_{i}-1)/2},$$

where *e*_*i*_ is the number of edges in the subgraph *G*_*i*_. The clustering coefficient of the network *C* is the mean of *C*_*i*_ of all the nodes in the network.

***Minimum path length.*** The nodal minimum path length *L*_*i*_ is defined as the mean shortest absolute path length of node *i* to other nodes in a network (Bullmore and Sporns, 2009), which quantifies the level of routing efficiency or the capability for parallel information propagation of a network (Van Den Heuvel et al., 2009; He and Evans, 2010; Liao et al., 2010)

$$L_{i}=\frac{1}{N-1}\sum_{i\neq j\in G}\min\{L_{i,j}\},$$

where min {*L*_*i,j*_} is the shortest absolute path length between node *i* and node *j*, and the absolute path length is the number of edges included in the path connecting two nodes. The characteristic path length *L* is the mean of *L*_*i*_ of all the nodes in the network.

***Efficiency.*** The nodal efficiency *E*_*i*_ is the inverse of the harmonic mean of the length between node *i* and all other nodes in the network, to deal with the disconnected graphs, non-sparse graphs or both (Latora and Marchiori, 2001; Bassett and Bullmore, 2006; Wang et al., 2010)

$$E_{i}=\frac{1}{N-1}\sum_{\begin{array}{l} j\in G\\ j\neq i \end{array}}\frac{1}{\min\{L_{i,j}\}}.$$

The global efficiency *E*_global_ of the network is the mean of *E*_*i*_ of all the nodes in the network.

In the subgraph *G*_*i*_, we can calculate the local efficiency of node *i* as:

$$E_{i\_\text{local}}=\frac{1}{N_{G_{i}}(N_{G_{i}}-1)}\sum_{\begin{array}{l} j,k\in G_{i}\\ j\neq k \end{array}}\frac{1}{\min\{L_{j,k}\}}.$$

The local efficiency *E*_local_ of the network is then similarly defined as the mean of *E*_*i*_local_ of all the nodes in the network (Rubinov and Sporns, 2010).

***Betweenness centrality.*** The betweenness centrality *BC*_*i*_ is defined as the fraction of all shortest paths in the network that pass through node *i* (Rubinov and Sporns, 2010). *BC*_*i*_ describes the central nodes that participate in many short paths within a network, and consequently act as important controls of information flow (Freeman, 1978)

$$BC_{i}=\frac{1}{\left(N-1)(N-2\right)}\sum_{\begin{array}{l} j,k\in G\\ i\neq j\neq k \end{array}}\frac{\rho_{j,k}(i)}{\rho_{j,k}},$$

where ρ_*j, k*_ is the number of shortest paths between node *j* and *k*; ρ_*j, k*_ (*i*) is the number of shortest paths between *j* and *k* that pass through node *i* (Rubinov and Sporns, 2010).

***Small-world parameters.*** Compared with random networks characterized by a low clustering coefficient and a typical short path length, networks with a small-world organization have a higher clustering coefficient and similar path length, i.e., γ = *C*/*C*_random_ > 1, λ = *L*/*L*_random_ ≈ 1, namely normalized clustering coefficient and normalized characteristic path length, respectively (Watts and Strogatz, 1998). These two conditions can also be summarized into a quantitative measurement, σ = γ/λ > 1, namely small-worldness (Humphries et al., 2006; Wang et al., 2010). *C*_random_ and *L*_random_ were calculated as the averaged clustering coefficient and characteristic path length of a set of 100 random networks with the same degree distribution as that of the examined functional connectivity network (Van Den Heuvel et al., 2009; Liao et al., 2010). The random networks were generated based on a Markov-chain algorithm, according to our previous study (Liao et al., 2010).

#### Threshold selection

The threshold *T* was defined as the total number of edges in a graph divided by the maximum possible number of edges (Achard and Bullmore, 2007), namely wiring cost. We investigated the topological properties of brain functional network over a range of *T*_min_ ≤ *T* ≤ *T*_max_. (1) *T*_min_ was selected by thresholding all networks to construct a sparse graph with the average degree *K* ≥ 2 × log (*N*) (here *N* = 90 represents the number of nodes); (2) *T*_max_ was selected to ensure the small-worldness σ of the thresholded networks be larger than 1.1 for all participants (Liao et al., 2010; Zhang et al., 2011). The resultant threshold range of 0.10 ≤ *T* ≤ 0.31 was used in our study. This range of sparsity allows the thresholded networks to be estimable for small-worldness and the number of spurious edges to be minimized (Watts and Strogatz, 1998; Achard and Bullmore, 2007; He et al., 2008; Zhang et al., 2011).

### Association between network organization and personality dimensions

All the nodal and global measures were thresholded repeatedly over the range of 0.1 ≤ *T* ≤ 0.31 with an interval of 0.01, and the area under the curve (AUC) for each network metric was calculated, which provides a summarized scalar for topological characterization of brain networks independent of single threshold selection (Zhang et al., 2011). The partial correlation was then calculated between the AUC of each network metric and extraversion/neuroticism scores, with age and gender being covariates.

To assess the statistical significance of the correlation, the null distribution for each network metric was obtained by non-parametric permutation tests. Accordingly, 5000 subject specific random networks were generated at each threshold as null-model reference networks. The correlations between the AUC of each network metric and the personality scores were recalculated to obtain the null distribution. 1/number of regions was used as a false-positive correction, which implied that there was less than one false positive regional result per cortical map at this threshold (Lynall et al., 2010; Fornito et al., 2011).

### Leave-one-out prediction

To test the validity of the significantly correlated measures in predicting personality scores of extraversion and neuroticism, a leave-one-out cross-validation strategy was applied. The significantly correlated measures acted as explanatory variables in the linear regression models to predict the personality scores. The predicted results of all the subjects were assessed by calculating the Pearson's correlation between the predicted values and the original values. The precision of individual prediction was assessed by the average of the absolute relative errors between the predicted and original scores.

## Results

### Descriptive statistics of the personality dimensions

Table 2 describes the scores of the three personality dimensions from the EPQ-RSC questionnaire, and Table 3 describes the correlations across the scores of the three dimensions. As two dimensions concerned in the present study, extraversion had a moderate negative correlation with neuroticism (*r* = −0.238, *p* = 0.046). The result was concordant with many prior studies, suggesting an inverse relationship between extraversion and neuroticism (Rusting and Larsen, 1997; Wright et al., 2006; Kim et al., 2008). Therefore, we added extraversion (or neuroticism) scores as covariate when calculating the partial correlation between neuroticism (or extraversion) and the AUC of each network metric, to obtain effects that were uniquely driven by each personality dimension.

**Table 2:** **Descriptive Statistics of the three personality dimensions of 71 participants**.

**Category**	**Data**
Gender (male/female)	38/33
Age (years)	23.219 ± 2.031
Extraversion (E)	56.172 ± 8.703
Neuroticism (N)	43.048 ± 12.822
Psychoticism (P)	46.581 ± 8.229

Age and personality scores are displayed as mean ± SD.

**Table 3:** **Correlations between scores of the three personality dimensions**.

	***p***	***N***
*N*	0.106 (*p* = 0.379)	
*E*	−0.205 (*p* = 0.086)	−0.238 (*p* = 0.046^*^)

*E, extraversion; N, neuroticism; P, psychoticism*.

* p < 0.05.

### The associations between network metrics and extraversion

**Figure 1:**
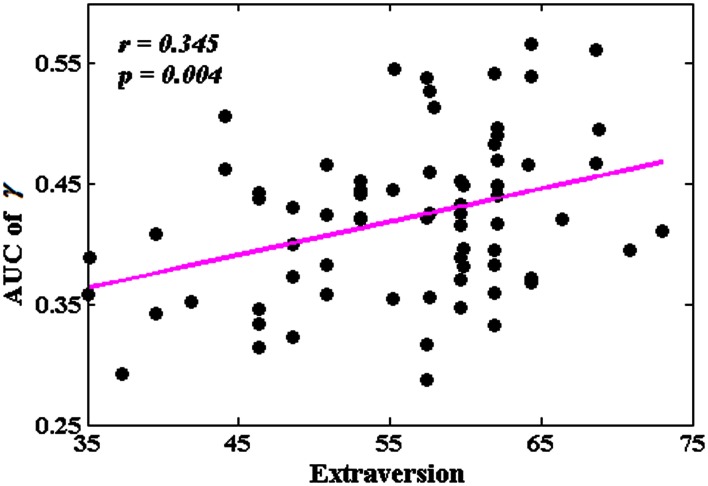
**The correlation between the area under the curve (AUC) of γ and extraversion scores (*p* < 1.90)**. AUC was calculated over the range of 0.1 ≤ *T* ≤ 0.31 with an interval of 0.01.

**Figure 2:**
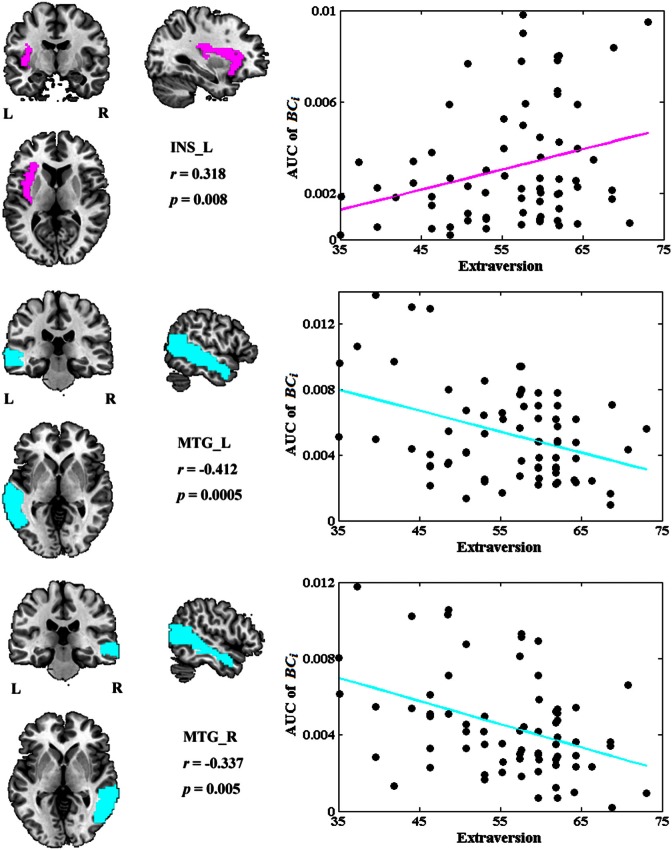
**The brain regions showing significantly correlations between AUC of *BC*_*i*_ and extraversion scores (*p* < 1.90)**. AUC was calculated over the range of 0.1 ≤ *T* ≤ 0.31 with an interval of 0.01. The cyan color represents the negative correlations, while the magenta color represents the positive correlations. INS, insular; L, left; MTG, middle temporal gyrus; R, right.

Among all the global measures of the network calculated in the present study, only the AUC of normalized clustering coefficient γ showed significant correlation with extraversion (Figure 1). As for the nodal measures, results indicated that only the AUC of *BC*_*i*_ showed significant correlations with extraversion. Extraversion significantly increased with *BC*_*i*_ in left INS, while significantly decreased with *BC*_*i*_ in bilateral MTG. Figure 2 demonstrates the brain regions showing significant correlations between their *BC*_*i*_ and extraversion scores along with the corresponding correlation coefficients.

**Figure 3:**
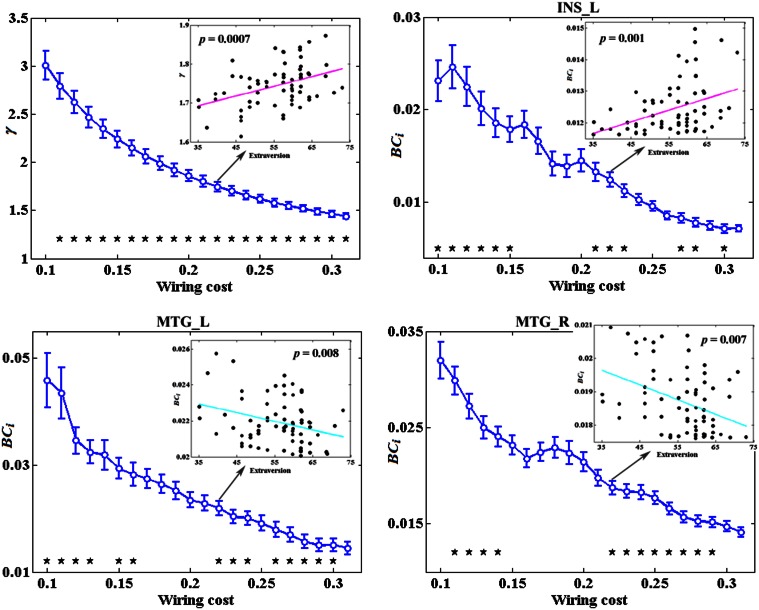
**The topological characteristics of network metrics which have significant associations with extraversion, as a function of wiring cost thresholds**. The asterisk indicates the threshold where the significant correlation between the metric and extraversion was detected (permutation testing, *p* < 1/90). The inset figure indicates the correlation between the metric and extraversion at wiring cost = 0.22.

Figure 3 depicts the topological characteristics of network metrics which have significant associations with extraversion, as a function of wiring cost thresholds. The asterisk indicates the threshold where the significant correlation between the metric and extraversion was detected (permutation testing, *p* < 1.90). The inset figure indicates the correlation between the metric and extraversion at wiring cost = 0.22.

### The associations between network metrics and neuroticism

**Figure 4:**
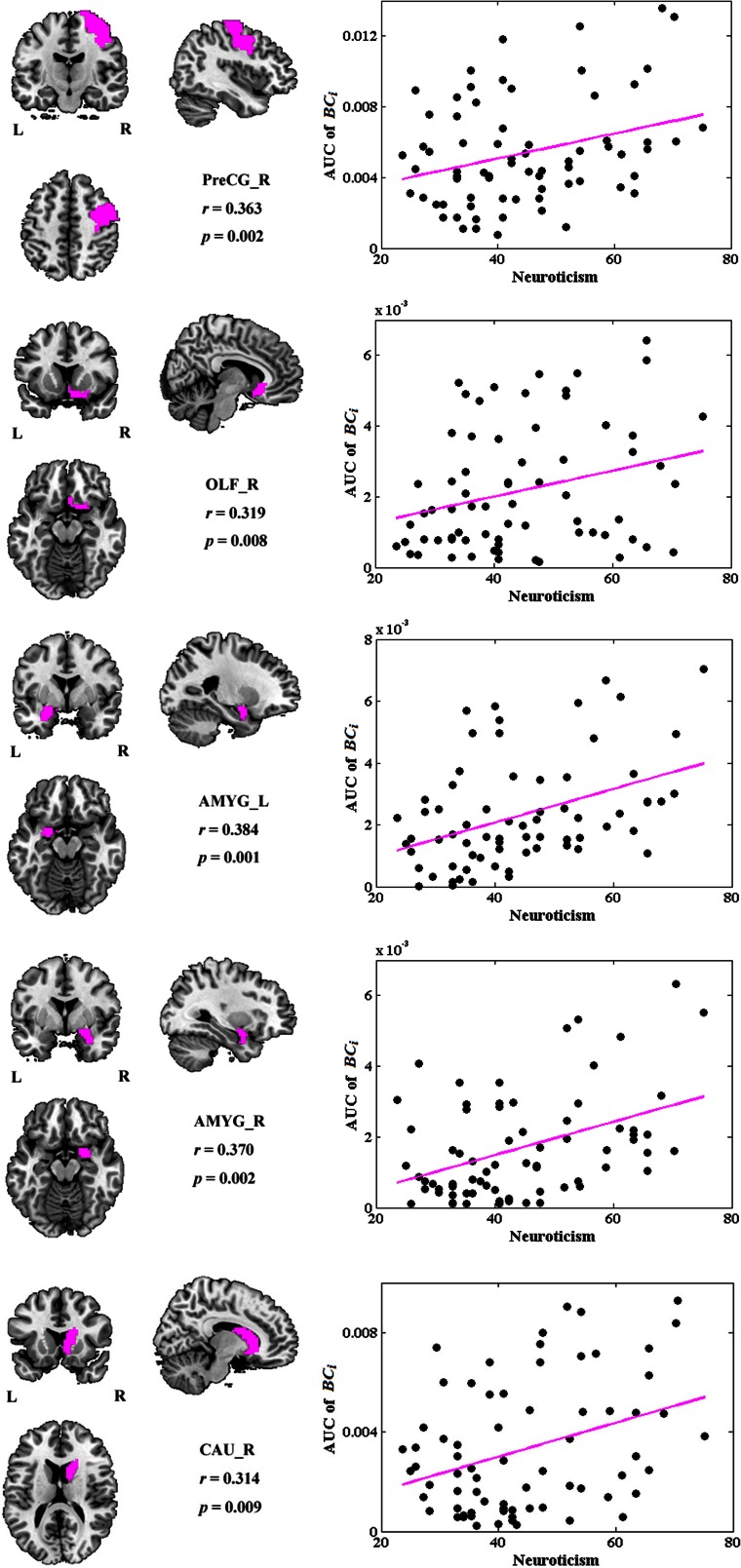
**The brain regions showing significantly correlations between AUC of *BC*_*i*_ and neuroticism scores (*p* < 1.90)**. AUC was calculated over the range of 0.1 ≤ *T* ≤ 0.31 with an interval of 0.01. AMYG, amygdala; CAU, caudate nucleus; L, left; OLF, olfactory cortex; PreCG, precentral gyrus; R, right.

No global measures showed significant correlation with neuroticism. Significant correlations were revealed in the AUC of *BC*_*i*_, too. Neuroticism scores showed increased significant correlation with *BC*_*i*_ in right PreCG, right olfactory cortex (OLF), right caudate nucleus (CAU), and bilateral AMYG. No significantly negative correlation was found. Figure 4 indicates the brain regions showing significant correlations between *BC*_*i*_ and neuroticism scores along with the corresponding correlation coefficients.

**Figure 5:**
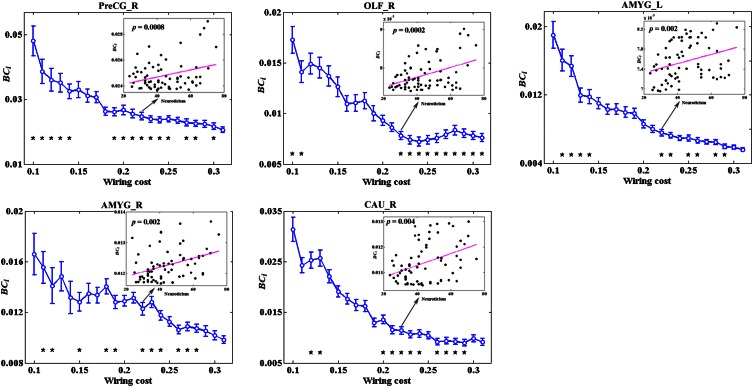
**The topological characteristics of *BC*_*i*_ as a function of wiring cost thresholds, in the brain regions whose *BC*_*i*_ values have significant associations with neuroticism**. The asterisk also indicates the threshold where the significant correlation between the metric and neuroticism was detected (permutation testing, *p* < 1/90). The inset figure indicates the correlation between the metric and neuroticism at wiring cost = 0.22.

Figure 5 depicts the topological characteristics of *BC*_*i*_ as a function of wiring cost thresholds, in the brain regions whose *BC*_*i*_ values have significant associations with neuroticism. The asterisk also indicates the threshold where the significant correlation between the metric and neuroticism was detected (permutation testing, *p* < 1.90). The inset figure indicates the correlation between the metric and neuroticism at wiring cost = 0.22.

### The prediction of personality scores by leave-one-out approach

**Figure 6:**
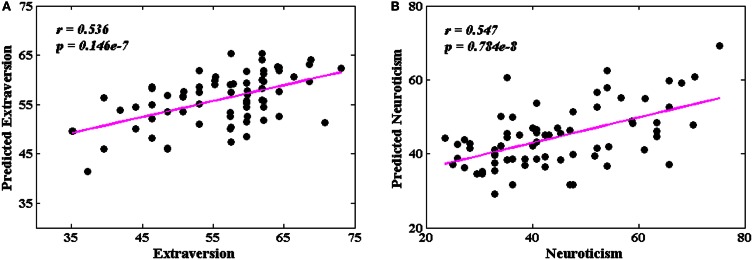
**The correlations between the predicted and original personality scores. (A)** Extraversion scores (*r* = 0.536, *p* = 0.146 × 10^−7^); **(B)** neuroticism scores (*r* = 0.547, *p* = 0.784 × 10^−8^).

Figure 6 shows the predicted and original pairs of extraversion (Figure 6A) and neuroticism (Figure 6B) scores, respectively. The Pearson's correlation coefficients of the predicted and original personality scores were 0.536 (*p* = 0.146 × 10^−7^) for extraversion, and 0.547 (*p* = 0.784 × 10^−8^) for neuroticism. The precisions of individual prediction were 11.4% for extraversion and 21.7% for neuroticism.

## Discussion

### Methodological considerations

The present study differed from our previous studies in both hypothesis and analysis methods. In the previous studies, the purpose was to identify the associations between the personality dimensions and the local synchronization of spontaneous BOLD activity (Wei et al., 2011), or between the personality dimensions and the fLFF in individual brain regions (Wei et al., 2012). Thereby the analysis method as well as the results obtained was at the functional segregation level.

Since the multiple spatially distinct brain regions are functionally connected with coherent temporal dynamics, the topological properties of the brain functional networks may predict individual differences in the two fundamental personality dimensions. To test this hypothesis, in the present study, we applied the graph theory method to explore the correlation between the network metrics in the resting-state brain network and the personality dimensions of extraversion and neuroticism at the functional integration level. To the best of our knowledge, the present study is among the first demonstrations of an association between personality dimensions and the properties of the resting-state functional network.

### Extraversion and the network metrics

The present results showed that compared to individuals with lower extraversion scores, individuals with higher extraversion scores had larger AUC of normalized clustering coefficient γ. γ quantifies the extent of local cliquishness or local efficiency of information transfer of a network (He and Evans, 2010). Our results indicated that the whole functional brain networks of individuals with higher extraversion scores were more clustered than these of individuals with lower extraversion scores.

In Eysenck's biological theory of extraversion, extraverts have a higher threshold for cortical arousal, as they are assumed to be chronically “under-aroused” (Eysenck, 1967; Tran et al., 2006; Wilt and Revelle, 2009). It is this chronic intrinsic under-arousal which is thought to drive highly extraverted people to engage in typically extraverted behaviors in order to enhance their low arousal states (Eysenck, 1994; Kehoe et al., 2012). Thereby extraverts should, on average, respond more and faster than introverts (in order to increase their arousal) during performance tasks (Eysenck, 1994; Wilt and Revelle, 2009). Furthermore, this under-arousability enables extraverts to tolerate much higher levels of arousal than introverts, who withdraw to avoid further increases in arousal which they find difficult to withstand (Eysenck, 1967, 1994; Kehoe et al., 2012).

From a network perspective, compared to random network which has low clustering coefficient and short path length, small-world network has higher clustering coefficient than a random network. The lower γ in the brain networks of introverts suggested that functional brain networks in introverts had a trend toward a randomized configuration. Random networks have less modularized information processing or fault tolerance compared with small-world networks (Latora and Marchiori, 2001; Zhang et al., 2011). A higher arousal threshold in cortex and higher level of arousal tolerance in extraverts could be interpreted by more clustered configuration in functional brain networks of extraverts.

As one of the nodal centrality metrics, increased *BC*_*i*_ suggested the strengthened roles of coordinating whole-brain networks (Zhang et al., 2011). The brain regions with relatively higher *BC*_*i*_ participate more in information transition and consequently act as important controls of information flow. The greater activation levels and lower arousal thresholds in these regions with higher *BC*_*i*_ may seem conceivable.

*BC*_*i*_ in left INS was found to be positively correlated with extraversion, whereas *BC*_*i*_ values in bilateral MTG were found to be negatively correlated with extraversion in this study.

INS is thought to play a central role for one's emotional processing (Iaria et al., 2008; Lamm and Singer, 2010) and is involved in various neuropsychiatric diseases such as mood disorders, depressive disorders, and panic disorders (Paulus and Stein, 2006; Nagai et al., 2007; Fitzgerald et al., 2008; Liu et al., 2010; Guo et al., 2011). There were many studies providing direct evidence for a link between INS activation and extraversion or extraversion-related dimension (Johnson et al., 1999; Omura et al., 2005a; Sollberger et al., 2009; Kehoe et al., 2012; Tamura et al., 2012). For example, a PET study showed the correlation between blood flow of INS and extraversion (Johnson et al., 1999). A fMRI study revealed that extraversion correlated with neural responses to positive word stimuli in bilateral INS (Omura et al., 2005a). A recent fMRI study suggested that INS activity may “mediate” the development of extraversion (Tamura et al., 2012). Our previous study found a positive association between extraversion and ReHo in INS. Positive correlation has also been found between extraversion and gray matter volume of INS in a morphometric study (Sollberger et al., 2009). The relation between *BC*_*i*_ and extraversion scores detected in INS in the present study further demonstrated that the regional characteristics of INS suggested individual differences in extraversion dimension. However, the positive correlation was unexpected according to Eysenck's predictions about this dimension. The expected negative correlations were revealed between extraversion and *BC*_*i*_ in bilateral MTG.

MTG was found to participate in both emotional processing and encoding tasks (Critchley et al., 2000; Dolcos et al., 2004; Olson et al., 2007). Abnormal functions of middle temporal areas in patients with borderline personality disorder (BPD) were reported both in resting MRI (Wolf et al., 2011) and emotional task MRI studies (Guitart-Masip et al., 2009). In normal late adolescents, fMRI study found correlations of age and extraversion with neural activation in MTG (Tamura et al., 2012). Our previous study also showed a negative association between extraversion and ReHo in MTG (Wei et al., 2011). These results demonstrated that MTG played a role in extraversion and the dysfunction of MTG was associated with personality disorders. Our study further showed the *BC*_*i*_ of bilateral MTG was negatively correlated with extraversion scores, suggesting that extraverts demonstrated lower levels of cortical arousal and higher arousal thresholds in bilateral MTG. This is consistent with Eysenck's arousal hypothesis of extraversion.

The results on the relationship between extraversion and arousal in specific brain regions some of which contradict Eysenck's predictions about this dimension while some of which agree with it have also been found in other studies (Kehoe et al., 2012; Wei et al., 2012). This may imply that the relationship between extraversion and arousal is a more complex story than that originally proposed by Eysenck (Kehoe et al., 2012). Researchers may face more complicated situations when looking into various brain regions. Further research is required to elucidate if and how arousal processing differences are a central feature of extraversion (Kehoe et al., 2012).

### Neuroticism and the betweenness centrality

The other main finding of this study was significantly positive associations between *BC*_*i*_ and neuroticism scores in brain regions including right OLF, right CAU, and bilateral AMYG.

According to Eysenck's biological theory of personality, high levels of neuroticism are theorized to reflect increased reactivity of the limbic system (Eysenck, 1991, 1994; Kehoe et al., 2012). Our results provided some supporting evidence for this hypothesis, with individuals high in neuroticism showing higher *BC*_*i*_ values in right OLF, right CAU and bilateral AMYG. The limbic system supports a variety of functions involving motivation, emotion, learning, and memory. Especially, AMYG has been associated with anxiety and mood disorders, for which neuroticism is a risk factor (Haas et al., 2007). Individual differences in neuroticism, a dimension characterized by experiences of negative emotion, anxiety, and emotional lability (Cunningham et al., 2010), have been shown to correlate with greater AMYG activation during unpleasant picture stimuli (Harenski et al., 2009), during fear learning (Hooker et al., 2008), during trials of high emotional conflict (Haas et al., 2007), and greater amygdala-dorsolateral prefrontal cortex connectivity while viewing angry and fearful facial expressions (Cremers et al., 2010). All these studies suggested a greater activation levels in AMYG during stimuli in individuals with high neuroticism, which were concordant with Eysenck's arousal theory of neuroticism. Here, our results further demonstrated that in resting-state functional brain networks, individuals high in neuroticism exhibited higher *BC*_*i*_ values in AMYG as well as in OLF and CAU, suggesting greater activation levels and lower arousal thresholds in these regions in the resting brains of high neuroticism subjects.

Interestingly, most significantly correlated brain regions were located in the right hemisphere, suggesting the lateralization of these regions with regard to neuroticism. Electroencephalogram (EEG) researchers have found that dimensions related to the withdrawal aspect of neuroticism are associated with greater activation of right frontal lobe relative to left (Zuckerman, 2005; Shackman et al., 2009). The brain's right hemisphere appears to be preferentially involved in emotions and motivational states associated with withdrawal, whereas left hemisphere is preferentially involved in approach (Davidson, 2002; DeYoung, 2010). Our results provided neurofunctional evidence from the point of view of functional brain networks in resting-state.

### The prediction of personality scores

In the present study, the leave-one-out cross-validation strategy was applied to test the validity of the significantly correlated measures in prediction personality scores. Our results showed very strong correlations between the original and predicted personality scores. The results suggested that statistically the functional brain network measures detected in our study did play roles in the prediction of personality. However, when considering the precision of individual prediction, the averages of the absolute relative errors for the prediction were 11.4% (range: 0.3–42.3%) for extraversion and 21.7% (range: 0.2–48.0%) for neuroticism. This implied the network measures alone were not able to precisely predict the individual personality. There have been some studies trying to predict individual personality from subjects' social behaviors (Bai et al., 2012) or emotional cognition tasks (Jackson, 2005). These studies combined with our findings shed light on the future work of precise prediction of personality, and further suggested that to integrate the information of individual's outer behaviors and inner topological properties of resting-state functional brain networks may give new clues to the neuropsychology of personality dimensions.

### Limitations

Since this was an exploratory study, no prior hypothesis for the relation of a certain measure to personality was proposed. The false-positive correction used in the present study, which was 1/number of regions, was not as conservative as a Bonferroni or false discovery rate (FDR) correction (Lynall et al., 2010). Thereby type I error was not able to be strongly controlled in our analyses. In addition, whether extraversion/neuroticism is correlated with intelligence is still under controversial. Studies on the Big five model of personality and intelligence found associations between extraversion/neuroticism and intelligence. Extraversion and intelligence were found to be significantly negatively correlated (Ackerman and Heggestad, 1997; Wolf and Ackerman, 2005); however, further study argued extraversion might be related to some aspects of intelligence test-taking, rather than to actual intelligence (DeYoung, 2011). Neuroticism exhibited a small but reliable negative correlation with intelligence (Ackerman and Heggestad, 1997) though; this correlation was likely to be due to the mediation by test anxiety (Moutafi et al., 2006). Higher-order traits may exist above the Big Five, but they do not appear to be related to intelligence (DeYoung et al., 2008). In Eysenck's personality theory, he asserted that intelligence is unrelated to personality (Eysenck, 1994). A study assessing personality by EPQ_R demonstrated that affective dimensions of personality are independent of intelligence (Gray et al., 2005). Since the present study aimed to test the Eysenck's personality theory of cortical arousal on extraversion and neuroticism, personality traits was asserted to be unrelated to intelligence. Further investigations of the relations between intelligence and extraversion/neuroticism are required in future studies.

## Conclusions

By applying the graph theoretical analysis to the resting-state fMRI data, the present study found that the normalized clustering coefficient values of the whole-brain functional networks were positively correlated with extraversion scores, suggesting an association between extraversion and the global network measure which quantifies the clustered configuration in the brain network. The more clustered configuration in brain functional network of extraverts may result in a higher arousal threshold in cortex and higher levels of arousal tolerance. However, extraversion scores were positively correlated with *BC*_*i*_ in right insula, while negatively correlated with *BC*_*i*_ in bilateral MTG, indicating that the relationship between extraversion and regional arousal is not as simple as that proposed by Eysenck. On the other hand, neuroticism scores showed consistently positive associations with *BC*_*i*_ in specific brain regions in the PreCG and limbic system, providing some supporting evidence for Eysenck's biological theory of neuroticism. Furthermore, the right lateralization of these regions with regard to neuroticism gave neurofunctional evidence to the preferential involvement of brain's right hemisphere in emotions and motivational states associated with withdrawal aspect of neuroticism.

### Conflict of interest statement

The authors declare that the research was conducted in the absence of any commercial or financial relationships that could be construed as a potential conflict of interest.
