# Erratum: Extraversion and neuroticism relate to topological properties of resting-state brain networks

**DOI:** 10.3389/fnhum.2013.00448

**Published:** 2013-08-06

**Authors:** Qing Gao

**Affiliations:** School of Mathematical Sciences, University of Electronic Science and Technology of ChinaCheng du, China

In the above paper, two mistakes were discovered after publication. The corrections are as following:

1. Zhiliang Long needs to be added as the third author.
2. All the “*p* < 1.90” in the paper should be corrected to “*p* < 1/90.”

